# Phosphonate coating of SiO_2_ nanoparticles abrogates inflammatory effects and local changes of the lipid composition in the rat lung: a complementary bioimaging study

**DOI:** 10.1186/s12989-018-0267-z

**Published:** 2018-07-16

**Authors:** Mandy Großgarten, Matthias Holzlechner, Antje Vennemann, Anna Balbekova, Karin Wieland, Michael Sperling, Bernhard Lendl, Martina Marchetti-Deschmann, Uwe Karst, Martin Wiemann

**Affiliations:** 1 Institute of Inorganic and Analytical Chemistry, University of Münster, Corrensstraße 28/30, 48149 Münster, Germany; 2 Institute of Chemical Technologies and Analytics, TU Wien, Getreidemarkt 9, 1060 Vienna, Austria; 3 IBE R&D Institute for Lung Health gGmbH, Mendelstraße 11, 48149 Münster, Germany

**Keywords:** Lung surfactant, Silica nanoparticles, In vitro and in vivo lung toxicity, MALDI-MS imaging, Phospholipids, Phosphonate coating, PI/PG ratio, Fourier transform infrared microspectroscopy imaging

## Abstract

**Background:**

The well-known inflammatory and fibrogenic changes of the lung upon crystalline silica are accompanied by early changes of the phospholipid composition (PLC) as detected in broncho-alveolar lavage fluid (BALF). Amorphous silica nanoparticles (NPs) evoke transient lung inflammation, but their effect on PLC is unknown. Here, we compared effects of unmodified and phosphonated amorphous silica NP and describe, for the first time, local changes of the PLC with innovative bioimaging tools.

**Methods:**

Unmodified (SiO_2_-n), 3-(trihydroxysilyl) propyl methylphosphonate coated SiO_2_-n (SiO_2_-p) as well as a fluorescent surrogate of SiO_2_-n (SiO_2_-FITC) nanoparticles were used in this study. In vitro toxicity was tested with NR8383 alveolar macrophages. Rats were intratracheally instilled with SiO_2_-n, SiO_2_-p, or SiO_2_-FITC, and effects on lungs were analyzed after 3 days. BALF from the right lung was analyzed for inflammatory markers. Cryo-sections of the left lung were subjected to fluorescence microscopy and PLC analyses by matrix-assisted laser desorption/ionization mass spectrometry imaging (MALDI-MS), Fourier transform infrared microspectroscopy (FT-IR), and tandem mass spectrometry (MS/MS) experiments.

**Results:**

Compared to SiO_2_-p, SiO_2_-n NPs were more cytotoxic to macrophages in vitro and more inflammatory in the rat lung, as reflected by increased concentration of neutrophils and protein in BALF. Fluorescence microscopy revealed a typical patchy distribution of SiO_2_-FITC located within the lung parenchyma and alveolar macrophages. Superimposable to this particle distribution, SiO_2_-FITC elicited local increases of phosphatidylglycerol (PG) and phosphatidylinositol (PI), whereas phoshatidylserine (PS) and signals from triacylgyceride (TAG) were decreased in the same areas. No such changes were found in lungs treated with SiO_2_-p or particle-free instillation fluid.

**Conclusions:**

Phosphonate coating mitigates effects of silica NP in the lung and abolishes their locally induced changes in PLC pattern. Bioimaging methods based on MALDI-MS may become a useful tool to investigate the mode of action of NPs in tissues.

**Electronic supplementary material:**

The online version of this article (10.1186/s12989-018-0267-z) contains supplementary material, which is available to authorized users.

## Background

Nanoparticles (NPs) consisting of silica are among the most common materials of everyday life. Besides other applications, silica NPs act as bulking agent in car wheels, as drug delivery system in cancer therapy or as food additive (E551) to prevent pulverulent foodstuffs from agglutinating. Respirable airborne silica may enter the lungs in special workplace situations and it is known for crystalline silica particles (quartz, cristobalite) that they elicit strong adverse health effects such as neoplastic transformation, progressive fibrosis or even cancer [1]. Amorphous nanosized silica particles are of less concern. They are produced by different production processes such that precipitated, fumed, or colloidal silica qualities are to be distinguished. From a toxicological point of view, most amorphous nanosized silica particles, at least above a certain dose, have been shown to cause acute pulmonary inflammation, but no progressive lung fibrosis [2–4]. Also, genotoxic or mutagenic effects, both of which had been described for cells in vitro mostly at high concentrations, have not been identified in the lung or secondary target organs [5]. This holds true also for comparatively high dose rates and even under conditions which increase the population of neutrophilic granulocytes inside the lung to extreme values [3].

The mechanisms underlying the cytotoxic, membrane disrupting or hemolytic potential of amorphous silica seem to involve silanol groups which are present at the particles’ surface and may interact with biological molecules such as proteins [6–9]. Accordingly, the biologic activity of amorphous silica NPs correlates largely with the overall size of the silica surface [10, 11] and modification of the chemical surface structures of silica NPs may alter their bioactivity. Effects of SiO_2_-n and SiO_2_-p used in this study have been investigated previously: coating of SiO_2_-n (diameter: 15 nm, BET surface: 200 m^2∙^g^− 1^) with 3-(trihydroxysilyl) propyl methylphosphonate (TPMP), which led to SiO_2_-p, largely abrogated the typical signs of inflammation elicited by the unmodified SiO_2_-n [12, 13]. The effect was not attributable to altered particle properties in general, as the TPMP coating had no or only minor effects on particle properties or agglomeration in biological media [12]. Interestingly, the in vitro binding of phospholipids from lung surfactant to SiO_2_-n and SiO_2_-p (both negatively charged) was similarly low, but increased in the presence of surfactant proteins A and D [14]. However, the binding of lung surfactant components to SiO_2_-n and SiO_2_-p under in vivo conditions has not yet been explored.

Lung surfactant is produced by alveolar type II cells and is released in the form of lamellar bodies which consist of 90% phospholipids and 10% surfactant proteins [15]. The unfolded lipid layer covers the inner surface of the lung and decreases the surface tension of the air-liquid interface [16]. The surfactant proteins A and D (so called collectins) are immunologically relevant as they can bind to microorganisms or foreign material such as (nano) particles, thus augmenting their uptake by alveolar macrophages [17–19]. For micron-sized crystalline silica and also for other particles it is known that they change the phospholipid composition (PLC) of the broncho-alveolar lavage fluid (BALF) in a time- and dose-dependent manner [20–22]: while the overall concentration of phosphatidylcholine (the major constituent of lung surfactant) increases, fractions of phosphatidylglycerol (PG) and phosphatidylinositol (PI) de- and increase, respectively [20]. Due to these opposed changes, the ratio PI/PG has been used as a sensitive tool to describe impairments of the lung. Of note, changes in PI/PG were observed during bleomycin- or quartz-induced lung fibrosis [20, 23], and also in humans suffering from the acute respiratory distress syndrome or other lung diseases such as cystic fibrosis [24, 25]. However, the impact of amorphous silica on the composition of the PLC of the lung is still unknown.

In contrast to previous studies on the composition of phospholipids in BALF, here we apply bioimaging methods to cryo-sections of the rat lung to demonstrate local lipid changes upon intratracheal instillation caused by amorphous silica NP in a laterally resolved manner. It is to be expected that allocation of changes in PI and PG to the presence of particles in the lung will improve our understanding of biological processes elicited by nanoparticles. To this aim, matrix-assisted laser desorption/ionization mass spectrometry imaging (MALDI-MS) and Fourier transform infrared (FT-IR) microspectroscopy imaging were applied to cryo-conserved lung sections to localize changes in the phospholipid composition secondary to the application of SiO_2_-n, SiO_2_-p and SiO_2_-FITC. With these methods, we found typical changes of distinct phospholipids to be co-localized with the distribution pattern of SiO_2_-FITC, whereas SiO_2_-p NP evoked no such changes.

## Results

### In vitro and in vivo toxicity study

**Fig. 1:**
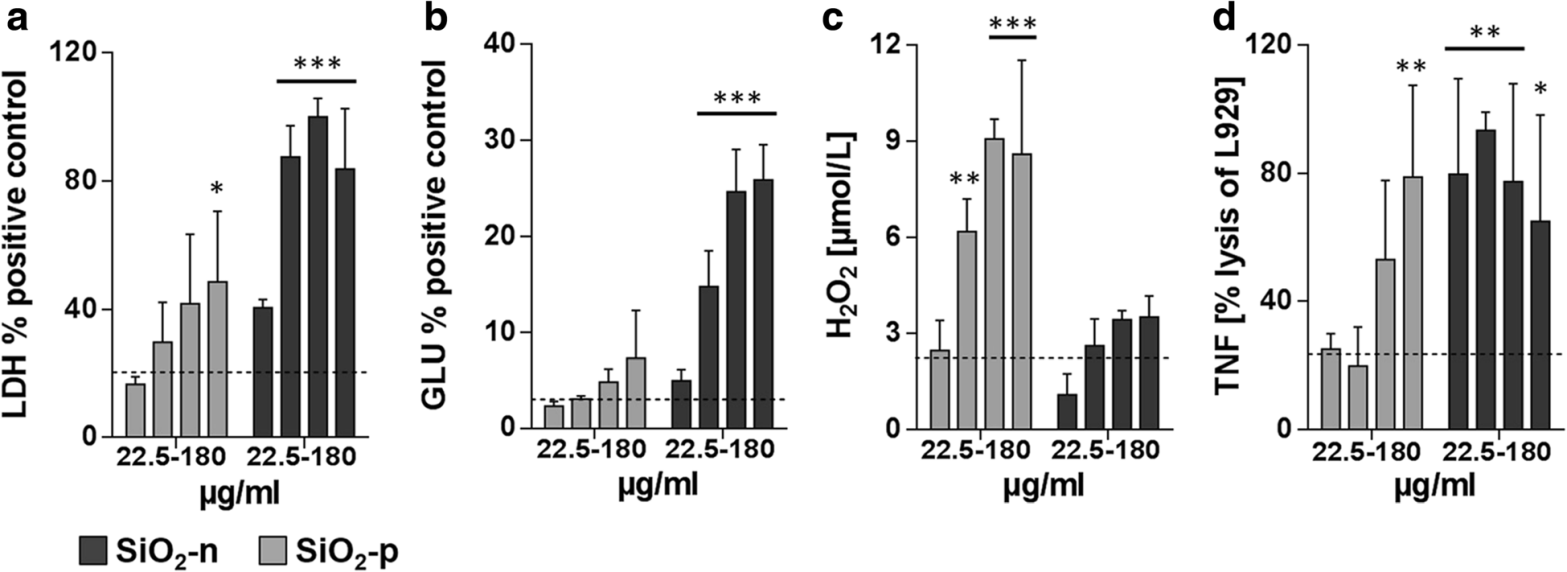
Effects of SiO_2_-n and SiO_2_-p NPs in vitro. **a**-**d** Dose dependent effects of SiO_2_-n and SiO_2_-p (22.5, 45, 90, and 180 μg·mL^− 1^) on NR8383 cells, taken from [54]. Release of **a** lactate dehydrogenase (LDH), **b** glucuronidase (Glu), **c** H_2_O_2_, and **d** tumor necrosis factor α (TNF-α). LDH and Glu activities were measured relative to positive control (Triton X-100-lysed cells) after 16 h. The H_2_O_2_ concentration was measured in μmol·L^− 1^ after 90 min, and bioactive TNF-α was measured after 16 h as lysis of TNF-α responsive L-929 cells. All columns represent mean values ± standard deviation of 3 independent experiments. Untreated cells served as controls (*n* = 3) whose mean values are indicated by dashed lines. Significance was tested by two-way ANOVA and post-hoc Dunnett’s multiple comparison test (*: *p* ≤ 0.05, **: *p* ≤ 0.01, ***: *p* ≤ 0.001)

To demonstrate the differential toxicity of the three SiO_2_ nanoparticle varieties, in vitro testing with a rat alveolar macrophage cell line was carried out with increasing concentrations of the particles (22.5, 45, 90, and 180 μg·mL^− 1^). Exposure to the pristine material SiO_2_-n for 16 h (Fig. 1a-d) led to dose-dependent increases in the cell culture supernatant of lactate dehydrogenase (LDH, a), glucuronidase (Glu, b), and TNF-α (d), mostly beginning at a concentration of 22.5 μg·mL^− 1^. These cytotoxic and inflammatory effects were far less pronounced upon SiO_2_-p, while the release of H_2_O_2_ from NR8383 cells, as measured during a 90 min incubation period, was augmented (c).

**Fig. 2:**
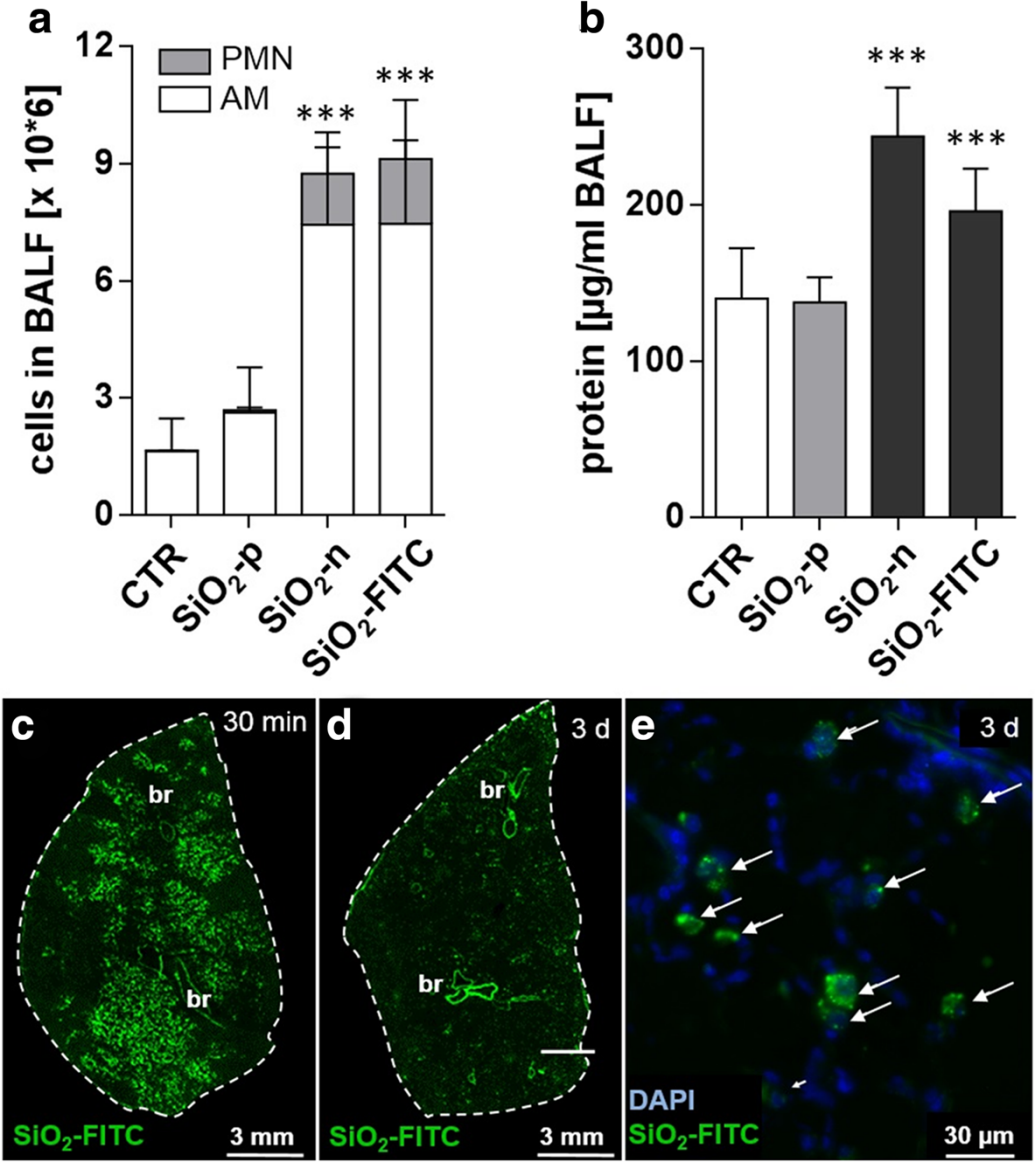
Effects of SiO_2_-n, SiO_2_-FITC, and SiO_2_-p in vivo and distribution of SiO_2_-FITC in the lung. SiO_2_-n_,_ SiO_2_-p, SiO_2_-FITC NPs were intratracheally instilled into rat lungs (0.36 mg per animal, *n* = 5 animals per group) and compared to vehicle-treated controls (CTR). **a**, **b** Analysis of broncho-alveolar lavage fluid 3 d post instillation: **a** alveolar macrophages (AM) and polymorphonuclear leukocytes (PMN); **b** total protein concentration. Columns represent means ± standard deviation; significance was tested by ANOVA and post-hoc Dunnett’s multiple comparison test (***: *p* ≤ 0.001). **c**-**e** Fluorescence micrographs of transversal cryo-sections of the left lung resected 30 min (**c**), and 3 d (**d**, **e**) after intratracheal instillation of SiO_2_-FITC. Dashed lines mark the outer rim of each section. Large bronchi (br) appear as strongly autofluorescent structures. **e** Detail of the section shown in (**d**); nuclei of lung cells were visualized with 4′, 6-diamidin-2-phenylindol (DAPI). Note that the fluorescent signal is confined to phagocytic cells (arrows) which were identified as alveolar macrophages in preceding studies

In vivo tests were carried out with SiO_2_-n, and SiO_2_-p. The fluorescence surrogate SiO_2_-FITC was used to demonstrate particle distribution in the left lung lobe secondary to intratracheal instillation which was carried out with a micro-sprayer device. In all tests, a concentration of 0.36 mg per rat lung was used to match the lung burden achieved in a previous inhalation study [13]. BALF analysis was conducted 3 days after intratracheal instillation of the NPs in order to examine their effect on cell counts and total protein content. SiO_2_-n and SiO_2_-FITC both increased the numbers of alveolar macrophages (AM) and polymorphonuclear leukocytes (PMN) as well as the concentration of total protein in BALF compared to the vehicle-treated control (Fig. 2a, b). Based on these biological effects, no difference was found between SiO_2_-FITC and SiO_2_-n NPs. Spray application of particles resulted in a patchy distribution pattern of particles inside the lung typically found upon intratracheal instillation (Fig. 2c) [26]. Higher magnification revealed many condensed fluorescent NP agglomerates alongside the alveolar septa after 30 min. After 3 d, the majority of this material had disappeared from alveolar walls but occurred within alveolar macrophages (Fig. 2d, e), whose overall distribution was still detectable by fluorescent microscopy and reflected the original sites of particle deposition. On hematoxylin-eosin stained lung cryo-sections SiO_2_-n or SiO_2_-p nanoparticle (or agglomerates thereof) were not detectable with bright field optics. However, SiO_2_-n or SiO_2_-FITC-treated lungs showed regions with increased macrophage numbers, slightly deteriorated structure and beginning hypercellularity. These changes were absent in lungs treated with SiO_2_-p and in vehicle-treated control lungs (Additional file 1: Figure S1).

### Identification of phospholipid species with MALDI-MS

**Fig. 3:**
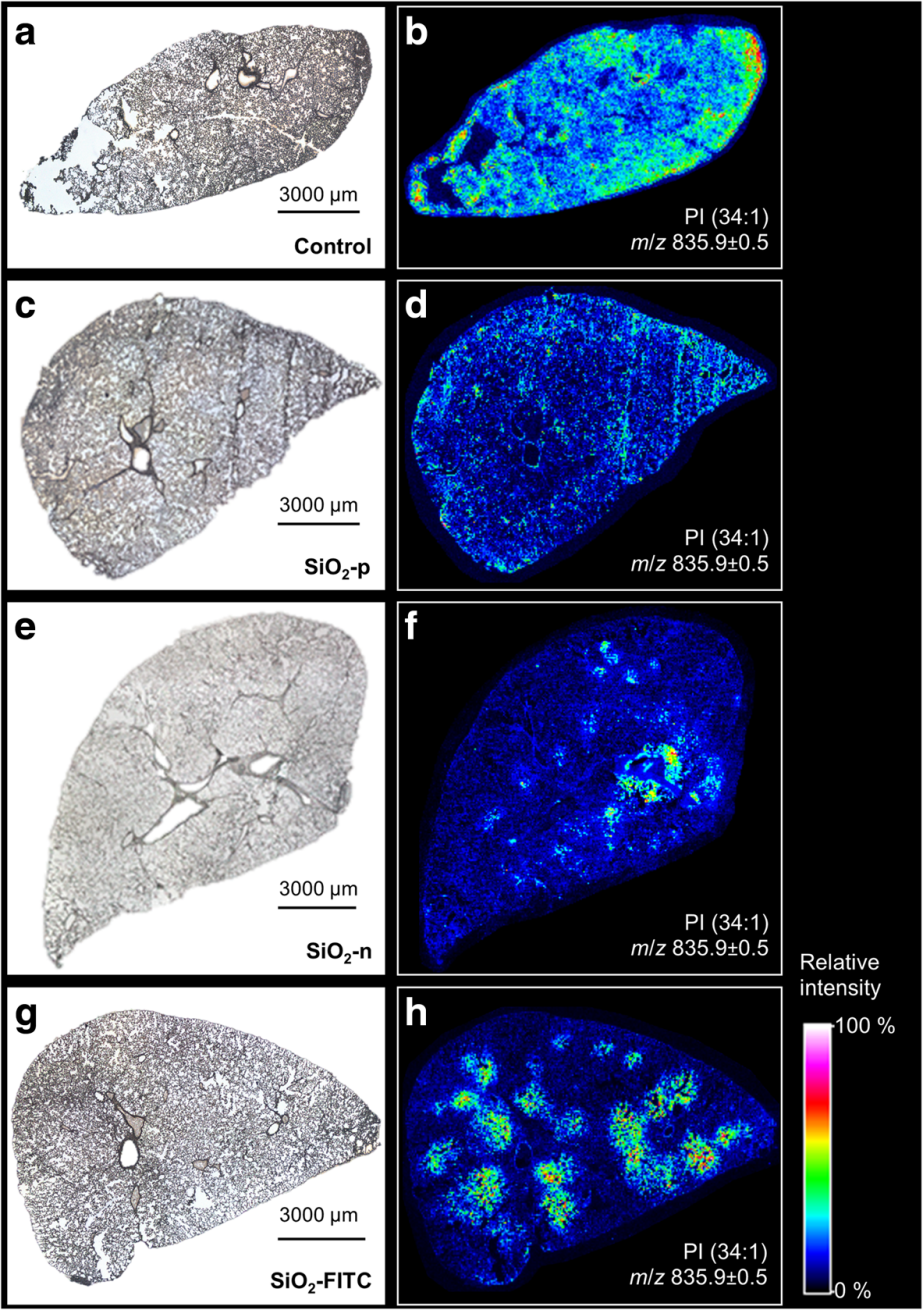
Local increases of PI concentration in the rat lung upon SiO_2_ NP treatment are abrogated by phosphonate coating. Distribution of phosphatidylinositol PI (34:1) 3 d after instillation of SiO_2_-n, SiO_2_-p, or SiO_2_-FITC (0.36 mg/lung): Microscopic images of the investigated cryo-sections (left) and corresponding MALDI-MS ion images of *m*/*z* 835.9 (right, detected as [M-H]^−^ in negative ion mode) of rat lung treated with **a**, **b** vehicle, **c**, **d** SiO_2_-p, **e**, **f** SiO_2_-n, or **g**, **h** SiO_2_-FITC. Note the patchy occurrence of PI upon SiO_2_-n and SiO_2_-FITC, but not upon SiO_2_-p. The seemingly high signal of the vehicle-treated control section results from automated scaling of the relative signal intensity

To get information about particle-related changes of the local PLC we analyzed representative cryo-sections of the lung from a vehicle-treated control animal for phospholipid distribution by MALDI-MS and secondary to the application of inflammatory SiO_2_-n or SiO_2_-FITC, as well as non-inflammatory SiO_2_-p NPs. Figure 3 shows low power micrographs of the sections and respective MALDI-MS ion images for mass-to-charge ratio (*m/z*) of 835.9 assigned to PI (34:1). This molecule gave a sufficient contrast in the negative ion mode and was selected as a starting point to highlight the distributional disparities between the three surveyed nanoparticle species. The lateral resolution (50 μm) of the method allowed to visualize major tissue components such as large and medium-sized bronchi.

The vehicle-treated control lung (Fig. 3a, b) exhibited a largely homogeneous distribution of PI (34:1) and all other detected *m*/*z* (Additional file 1: Figure S4). Signal inhomogeneity in this case was attributable to compression artifacts (lower right margin) or to a partial loss of the tissue (left hand side), as was evident from the low-power micrograph (Fig. 3a). Of note, as the pseudocolor scale reflects relative intensity values and spreads the complete set of data from 0 to 100%, a comparison of absolute values cannot be made for different treatments, i.e., between different tissue sections. Yet, absolute intensity values within one tissue section can be compared.

Particle treatment could change the homogeneous lipid distribution pattern in a striking manner: While SiO_2_-p had no influence on the lipid distribution (Fig. 3d), SiO_2_-FITC NPs induced round-shaped patchy regions within which the PI (34:1) signal was strongly increased (Fig. 3h). Similarly, but with larger heterogeneity, SiO_2_-n induced several regions with increased PI (34:1) (Fig. 3f).

**Fig. 4:**
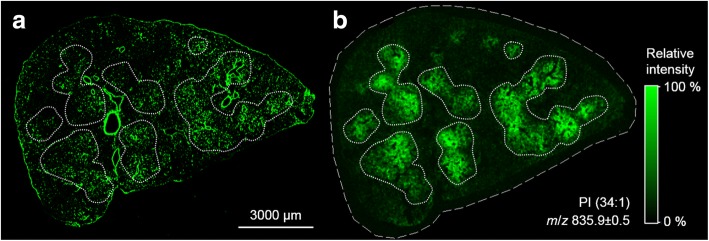
Correlation of nanoparticle distribution and local PI overexpression. Cryo-section 3 d post instillation of SiO_2_-FITC NPs. **a** Fluorescence overview image visualizing the principal distribution of NPs mainly located in alveolar macrophages. **b** MALDI-MS ion image of PI (34:1) ([M-H]^−^) in a parallel tissue section (bordered by a dashed line). The fine dotted lines demarcate seven main areas of PI overexpression. These areas were transferred to the fluorescence image in (**a**) to show the co-localization with FITC fluorescence. Large blood vessels and bronchioli show strong autofluorescence, but no PI signal

To analyze whether there is a congruency of particle distribution and the pattern of increased PI (34:1) intensity, we compared serial sections of the same lung for their distribution patterns of FITC fluorescence and PI (34:1). Figure 4 shows the overview distribution of FITC fluorescence and the PI (34:1) signal: Hand-drawn regions demarcating PI (34:1)-enriched regions were transferred from Fig. 4b to the fluorescent image of Fig. 4a. Although connective tissue around bronchiolar structures and blood vessels stands out clearly due to considerable autofluorescence, fluorescent dots in PI (34:1)-enriched regions are far more numerous. At higher magnification these fluorescent signals represent SiO_2_-FITC-laden cells (compare Fig. 2e) which have gathered the fluorescent material.

**Fig. 5:**
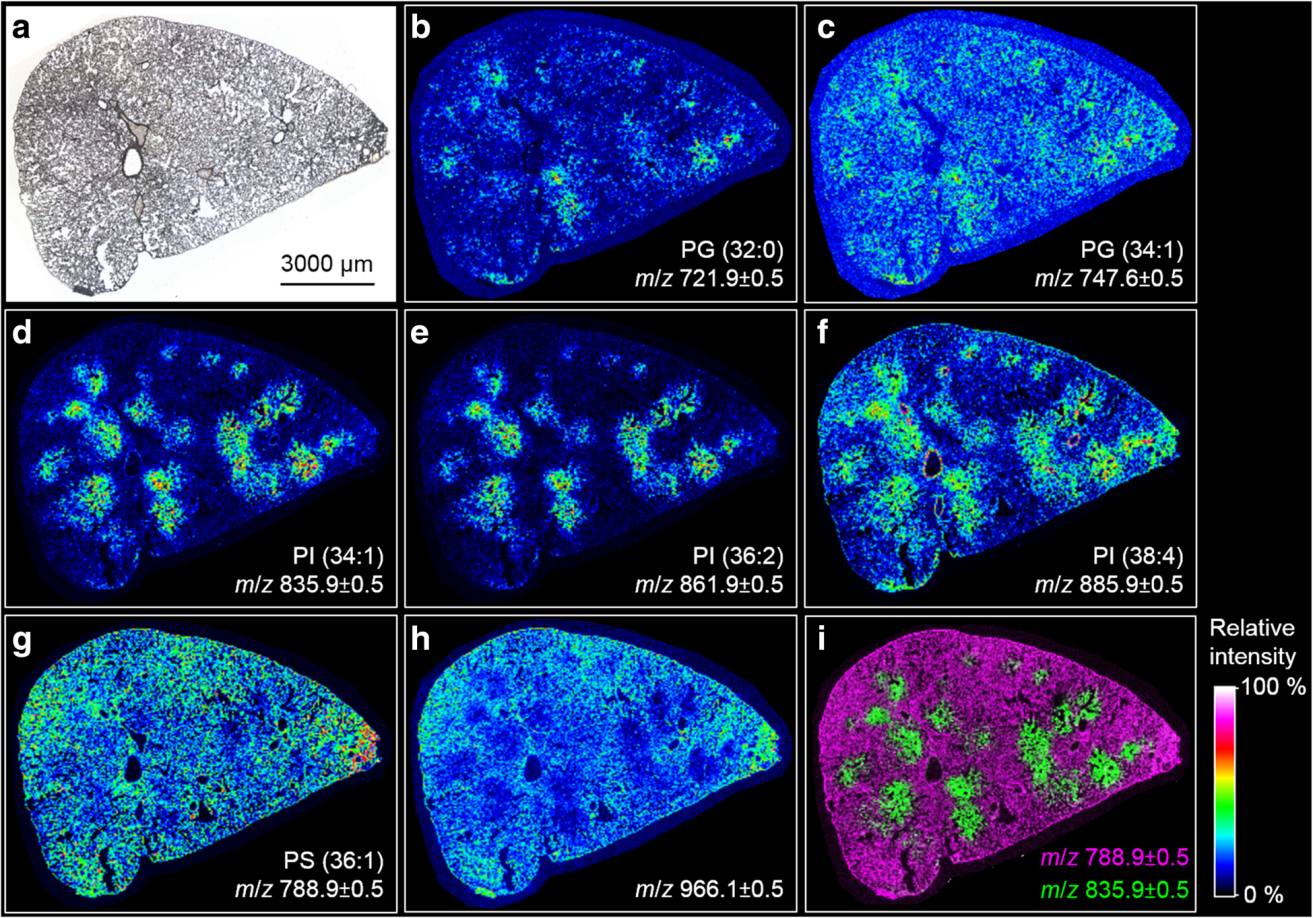
MALDI-MS ion images of local changes of lipid concentration in the SiO_2_-FITC laden rat lung. Cryo-section from an animal 3 d after intratracheal instillation of 0.36 mg SiO_2_-FITC NPs. **a** Microscopic image of investigated cryo-section. **b**, **c** Ion images indicating a minor local overexpression of PGs. **d**-**f** Ion images with pronounced local overexpression of PIs. **g**, **h** Ion images of *m*/*z* 788.0 ± 0.5 and 966.1 ± 0.5 revealing an inversely correlating distribution of PS and TAG compared to the PIs shown in (**d**, **e**) and (**f**). **i** Overlay of MS ion images shown in (**d** and **g**); *m*/*z* are assigned to ion [M-H]^−^

In the next step we imaged further (phospho) lipids and analyzed if distribution changes were congruent to the patchy PI (34:1) signal elicited by SiO_2_-n and SiO_2_-FITC. These in-depth analyses were carried out on sections of lungs instilled with SiO_2_-FITC (Fig. 5), particle-free instillation fluid (Additional file 1: Figure 4S), and SiO_2_-p (Additional file 1: Figure 5S). Figure 5 displays the MALDI-MS ion images of distinct *m*/*z* representing a variety of phospholipids detected in negative ion mode in the lung section originating from a rat instilled with SiO_2_-FITC NPs. Detected *m*/*z* were classified as phosphatidylglycerol (Fig. 5b, c), phosphatidylinositol (Fig. 5d-f) and phosphatidylserine (PS) (Fig. 5g), each featuring two fatty acyl residues. The assignment of *m*/*z* 966.1 (Fig. 4h) as triacylglycerine (TAG) will be discussed below. Shorthand designations, which will be used in the next section, represent the length of the carbon chains summarizing all fatty acyl residues and the degree of unsaturation, i.e. the number of double bonds within fatty acid chains. Most striking, the round-shaped regional overexpression of the previously mentioned PI (34:1) with a *m/z* of 835.9 (Fig. 5d) was largely colocalized with *m*/*z* which can be assigned to [M-H]^−^ ions of PI (36:2) (*m*/*z* 861.9), and PI (38:4) (*m*/*z* 885.9, Fig. 5e, f). Interestingly, highest concentration of PI (38:4) apparently lined the larger bronchi. The pattern of round-shaped regional increases was found, to a lesser extent, also for *m*/*z* corresponding to phospholipids of the phosphatidylglycerol (PG) class (Fig. 5b, c), such that the overexpression patterns of PIs and PGs were highly co-located.

Compared to PIs and PGs, a mild inversely correlating distribution was found for *m*/*z* 788.9 assigned to PS (36:1) (Fig. 5g), and in an even more pronounced manner also for *m*/*z* 966.1 (Fig. 5h). The contrastive distribution of different phospholipids is further shown in an overlay image of *m*/*z* 788.9 and *m*/*z* 835.9 (Fig. 5i).

For verification purposes, two exemplary *m*/*z*, namely, *m*/*z* 721.4 assigned to PG (32:0) and *m*/*z* 861.5 assumed to be PI (36:2) were selected as precursor ions for tandem mass spectrometry (MS/MS) experiments. Mass spectra of their characteristic fragments (Additional file 1: Figures S2 and S3) confirm the abundance of PIs and PGs as the major phospholipid classes detected in the negative ion mode. They further indicate that PG (32:0) consists of two (16:0) chains (Additional file 1: Figure S4), while for PI (36:2) both fatty acyl compositions, PI (18:1|18:1) and PI (18:0|18:2), are deduced (Additional file 1: Figure S5).

Since especially the ratio PI/PG is a well-known marker of lung affection in BALF, we calculated a local PI/PG on the basis of absolute intensity data. Regions of interest (ROIs) were defined on sections of SiO_2_-FITC-treated and vehicle-treated lungs, within which the signal intensities of PI (34:1) (*m*/*z* 835.9) and PG (34:1) (*m*/*z* 747.6) were integrated from 600 spectra. On an untreated lung tissue section, where phospholipids appeared evenly distributed, a control ROI was chosen randomly. Its absolute intensity values were 0.67 a.u. (arbitrary units) for PI (34:1) and 0.36 a.u. for PG (34:1), resulting in a PI/PG of 1.9. On a lung section from a SiO_2_-FITC instilled animal, a ROI with an apparent local PI overexpression was chosen. The absolute intensities therein were calculated as to be 2.15 a.u. for PI (34:1) and 0.38 a.u. for PG (34:1) resulting in a high local PI/PG ratio of 5.7. Together with the localization of SiO_2_-FITC the result shows that the PI/PG was locally increased in SiO_2_-FITC-laden regions.

**Fig. 6:**
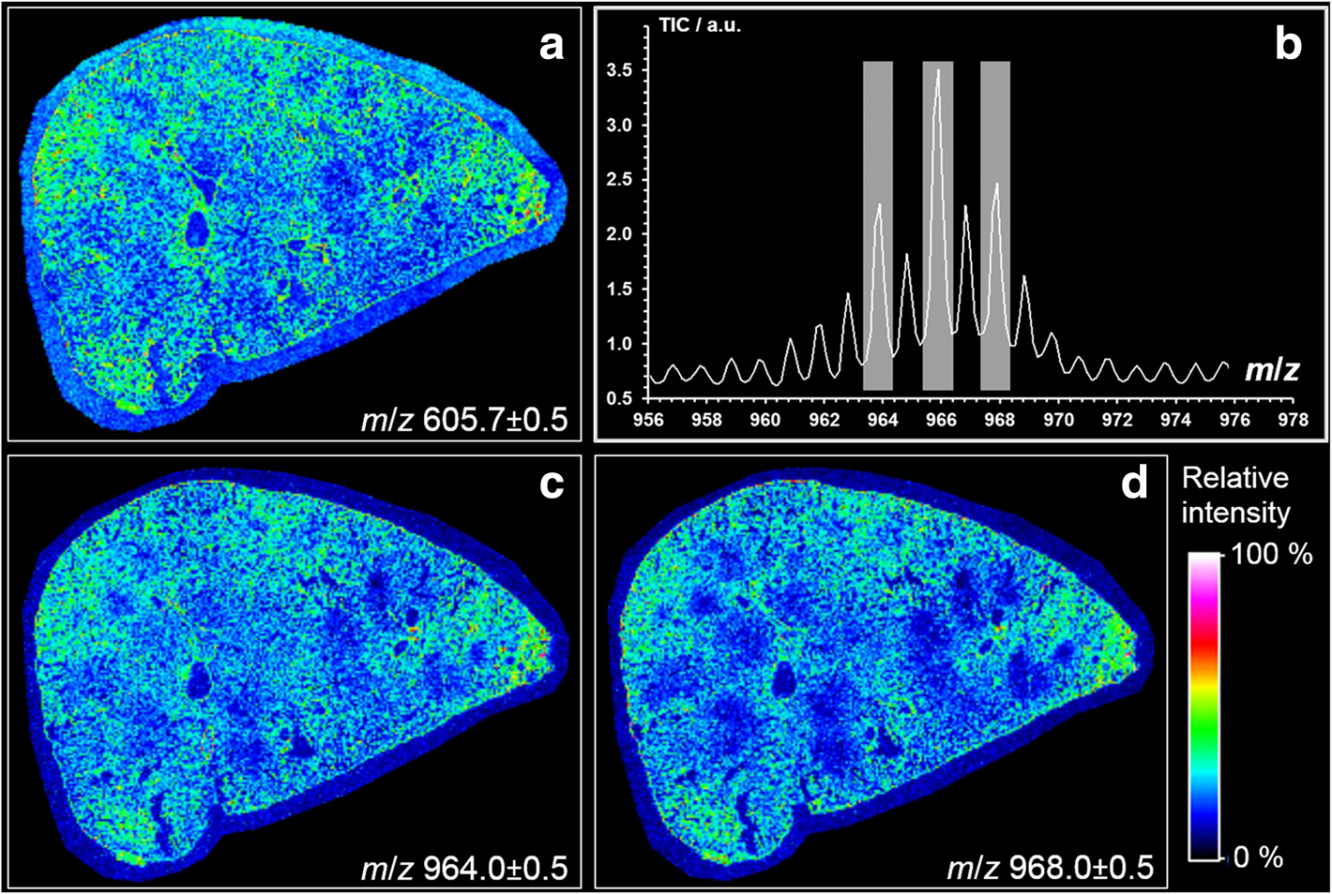
MALDI-MS ion images showing the local distribution of TAG and DAG-like molecules in a SiO_2_-FITC laden rat lung. Cryo-section from an animal 3 d after intratracheal instillation of 0.36 mg SiO_2_-FITC NPs. Ion images of **a** *m*/*z* 605.7 from DAG-like fragment (36:1) as [M–H_2_O + H]^+^ detected in the positive ion mode, **c** *m*/*z* 964.0 assumed to be TAG (60:5), **d** *m*/*z* (968.0) assumingly from TAG (60:3). **b** Relevant section of the average MALDI-MS spectrum (negative ion mode) of the analysis of the rat lung tissue shown in (**c** and **d**)

In contrast to PI and PG, we found *m*/*z* 966.1 and other closely related masses to be lowered in particle-laden regions (Fig. 5h). Results obtained by MALDI-MS suggest that *m*/*z* 966.1 represents a triacylglyceride TAG (60:4). Interestingly, the signal was co-distributed with closely related molecules whose peaks showed mass differences of two mass units (*m*/*z* 964.0, 966.0 and 968.0, see Fig. 6) and, therefore, might correspond to related TAGs whose number of double bonds range from 3 to 5. Further evidence for the correct detection of TAG comes from the co-distribution of a DAG-like derivative *m*/*z* 605.7 (Fig. 6a) because this fragment is derived from TAG in tissue by cleavage of a fatty acyl residue [27].

None of the aforementioned patchy irregularities of the PLC was observed in the vehicle-treated or SiO_2_-p-treated animals, as is shown in Additional file 1: Figures S4 and S5 of the Supplementary Information.

### Fourier transform infrared microspectroscopy and hierarchical cluster analysis

**Fig. 7:**
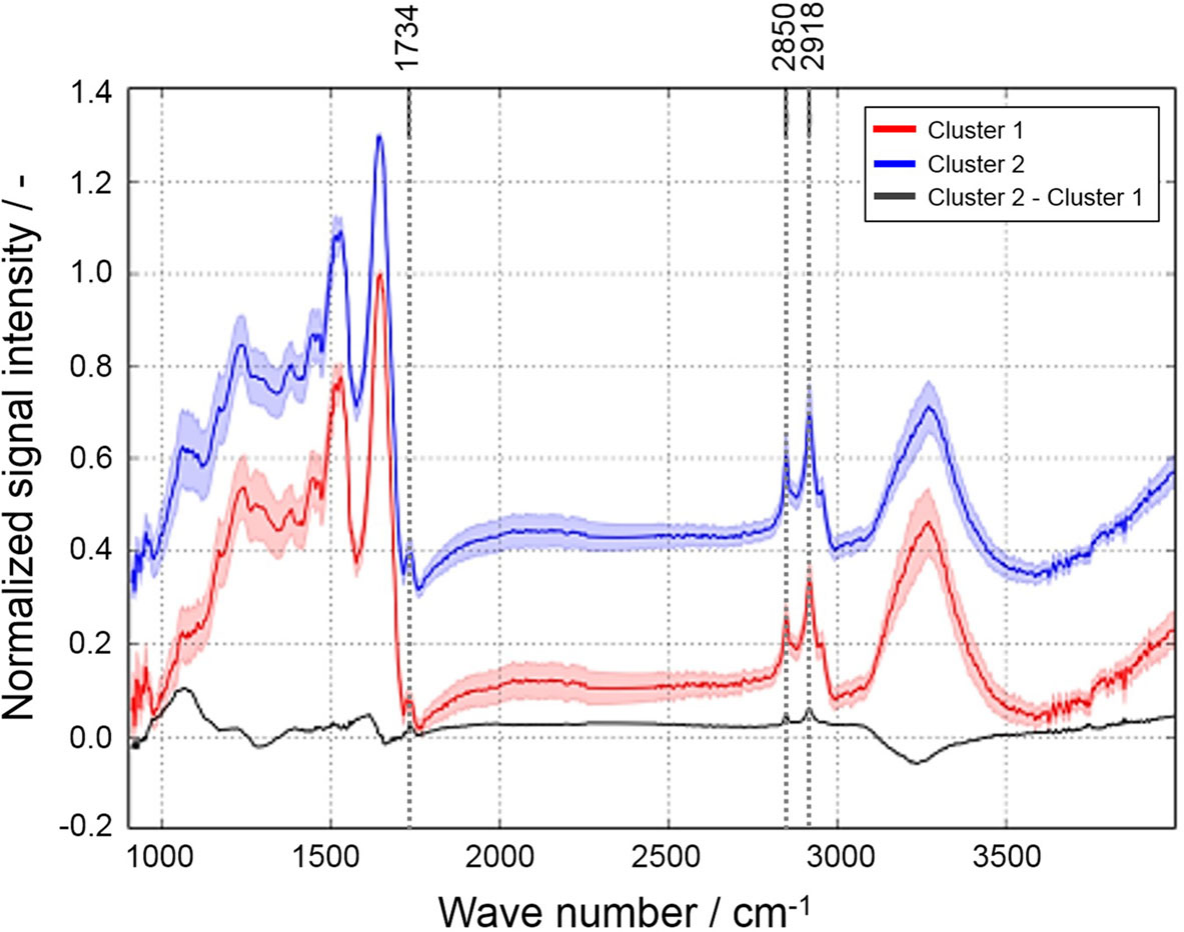
FT-IR microspectroscopy of a cryo-section from a SiO_2_-FITC-laden rat lung. Average spectra and standard deviation from hierarchical cluster analysis. Red spectrum: cluster 1; blue spectrum: cluster 2 (an offset was added to cluster 2 for better visualization); black spectrum: difference spectrum (cluster 2 - cluster 1). Dashed lines mark wavenumbers identifying lipids. Protein bands are highlighted in grey

Unlike MALDI-MS Fourier transform infrared (FT-IR) microspectroscopy provides molecule specific information based on the investigation of spectral fingerprints consisting of vibrations which can be assigned to functional groups of the building blocks of biological tissues, such as lipids, proteins, carbohydrates, and nucleic acids. To test whether lipid-enriched areas seen with MALDI-MS could be verified with another independent technique, FT-IR microspectroscopy was carried out on parallel sections of the SiO_2_-FITC laden rat lung tissue, i.e. adjacent to the sections investigated with MALDI-MS. The pre-processed spectral data generated by means of FT-IR imaging were subjected to a hierarchical cluster analysis (HCA), which is a powerful tool to statistically validate the spectral disparities between the pixels within an image. Figure 7 shows average spectra of two ascertained clusters (cluster 1: red line; cluster 2: blue line) along with their difference spectrum (cluster 2 - cluster 1: black line). Three positive deviations at defined position of this difference spectrum (arrows in Fig. 7) indicate that the blue cluster pixels have more intense vibrations resulting from lipids (CH_2_ asymmetric stretching vibration at 2918 cm^− 1^ and CH_2_ symmetric stretching vibration at 2850 cm^− 1^) and esters of free fatty acids (C=O stretching vibration at 1734 cm^− 1^). In contrast, the red cluster pixels show increased signal intensities for bands in the spectral ranges of 1695–1620 cm^− 1^, 1580–1480 cm^− 1^, and 3290 cm^− 1^ (grey areas in Fig. 7) which can be assigned to peptide groups termed Amide I, Amide II and Amide A, respectively. It can thus be concluded that the red cluster 1 indicates areas of higher protein signal, while the blue cluster 2 corresponds to areas with higher lipid content.

**Fig. 8:**
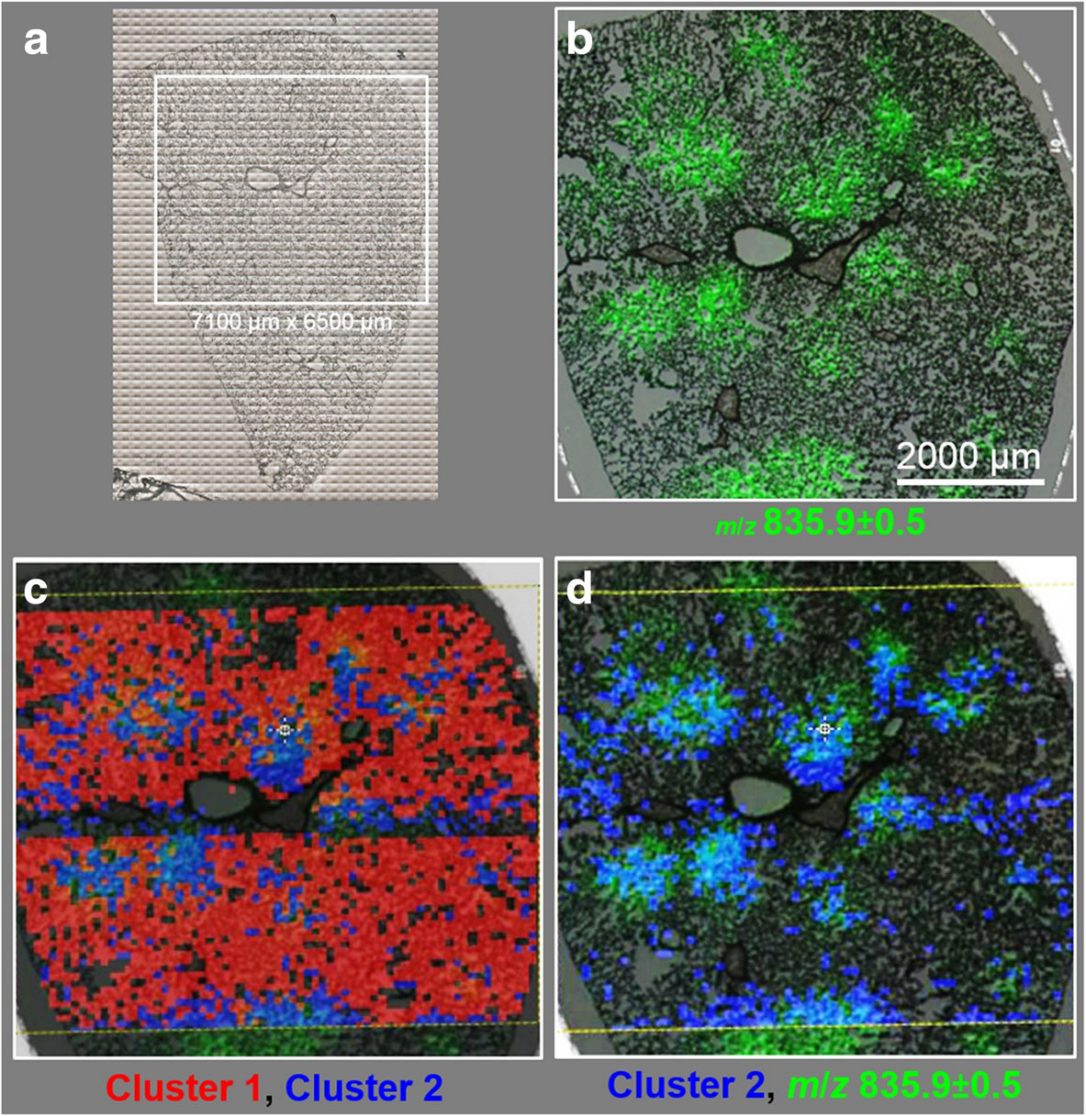
Comparison of MALDI-MS imaging with FT-IR spectroscopy followed by add-on hierarchical cluster analysis. **a** Optical image of lung cryo-section. Region inside the white box was analyzed by means of FT-IR imaging. **b** Overlay of optical image with MALDI-MS ion image of PI 34:1 of the marked area. **c** Overlay of (**b**) with cluster 1 (red) and cluster 2 pixels (blue), as derived from HCA of FT-IR data analysis (Fig. 6). **d** Overlay of (**b**) with cluster 2 pixels (blue), indicating that their positions are largely co-located with regions of PI overexpression

Figures 8a-d show the superposition of the spatially resolved images of clusters 1 and 2 to the MALDI-MS image of PI (34:1, *m*/*z* 835.9), and the optical image of the SiO_2_-FITC laden lung tissue section from Fig. 8b. Comparing the color-coded regions it can be seen that the lipid-enriched cluster 2 pixels (blue) were largely co-localized with PI (34:1) (Fig. 8d), whereas protein-enriched cluster 2 pixels (red) were evenly distributed throughout the lung parenchyma but were lowered in PI (34:1) enriched regions (Fig. 8c). Thus, the detection of lipid enrichment by FT-IR microspectroscopy confirms results from MALDI-MS studies on adjacent sections.

## Discussion

This study has shown that the cytotoxicity and acute inflammation typically induced by SiO_2_-n or SiO_2_-FITC NP was not elicited by SiO_2_-p, whose surface is modified by phosphonate residues. The primary distribution of SiO_2_-FITC administered to the lung was mirrored by local changes of PI, PG, PS and TAG as measured for the first time by MALDI-MS and confirmed by FT-IR spectroscopy. A similar patchy distribution of phospholipids was obtained in SiO_2_-n-treated, but not in vehicle-treated lungs. Importantly, SiO_2_-p evoked no such changes in the local (phospho) lipid composition pattern. Together the findings suggest that alterations in PLC were secondary to local inflammatory processes.

### Effects of phosphonate coating on bioactivity of SiO_2_

The cytotoxic, membrane disrupting and/or hemolytic potential of amorphous silica has been attributed to silanol groups at the particle surface [6–9]. Pandurangi et al. observed a correlation between the concentration of surface silanol groups determined by means of FT-IR spectroscopy and the hemolytic activity of silica particles expressed as enhanced cell lysis of sheep blood erythrocytes [8]. Adverse effects of silica may, therefore, be reduced by modifying surface silanol groups, as shown for cristobalite which lost its cytotoxicity upon heating to 1300 °C, a treatment which condenses silanol groups to siloxane bridges [9]. Cytotoxic and inflammatory effects of silica may also be suppressed by coating with hydrophobic substances [28], polyvinylpyridine-N-oxide [29], or amino groups [12]. As the TPMP coating of SiO_2_-n, which generated the SiO_2_-p used in this study, largely reduced cytotoxic and inflammatory properties, phosphonate residues appear well suited to protect cell and tissue components against effects of silanol groups or other types of surface reactivity. Interestingly, a reduction of particle reactivity in vitro and in vivo has also been achieved for NP composed of rare earth elements [30] or of partially soluble metals such as Ni, Co, and Cu [31] using ethylenediamine tetra (methylene phosphonic acid) (EDTMP) as a coating agent. However, EDTMP can chelate metal ions released from NP surface [31] and this mode of action may underlie the beneficiary effect of EDTMP which, therefore, differs from that of TPMP. Nevertheless the outwardly directed phosphonate residues of both, EDTMP and TPMP, seem to confer a high degree of biocompatibility to NPs.

Although phosphonate coating lowered the cytotoxicity of SiO_2_-n, SiO_2_-p dose-dependently increased the release of H_2_O_2_ from alveolar macrophages in vitro (Fig. 1c). Similar to primary alveolar macrophages, NR8383 cells respond to specific stimuli such as the non-cytotoxic zymosan with an oxidative burst [32]. However, the mechanism underlying the augmented induction of H_2_O_2_ by SiO_2_-p is unknown. At least for high concentrations of SiO_2_-n (and also for other nano-sized amorphous silica materials, own unpublished observations) there is a tendency to induce a release of H_2_O_2_ from NR8383 cells but this effect may be counteracted by the cytotoxicity of SiO_2_-n under serum-free conditions. The enhanced formation of H_2_O_2_ upon SiO_2_-p may, therefore, be favored by the low cytotoxicity of the phosphonated material, although a more direct stimulation of H_2_O_2_ generating processes cannot be excluded. Of note, the comparatively low dose of SiO_2_-p had no obvious effect on the lung as it elicited neither signs of tissue damage, nor did it increase inflammatory cell counts in the lavage fluid.

### Methodological considerations of MALDI-MS

MALDI-MS is most commonly used for the spatially resolved determination of biomolecules such as lipids and proteins as well as drugs and their metabolites [33–35]. Due to fast laser scan speed and high sensitivity, while covering a broad mass range, MALDI-MS provides high potential for the determination of lipids and has successfully been applied to study the composition of broncho-alveolar lavage fluid [36, 37]. In the lung a fairly even distribution of phospholipids has been shown for the lung parenchyma by MALDI-MS, whereas there was a differential overexpression of arachidonate/docosahexaenoate phospholipids and sphingomyelin molecular species lining the profiles of larger bronchioli and blood vessels, respectively [35].

Surprisingly, no information is available on the influence of nanoparticles on the spatial distribution of phospholipids in lung tissue [35]. By weight, ~ 90% of the lung surfactant consists of lipids, from which phosphatidylcholine (PC) is the major component (70–80%). In addition, variable amounts of phosphatidylglycerols (7–18%), phosphatidylinositols (2–4%), and phosphatidylethanolamines (2–3%) are contained [38]. As the MALDI-MS analyses presented here were conducted in the negative ion mode, acidic phospholipids such as the low abundance PIs and PGs are preferably detected and this is in contrast to PCs, which are rather ionized in positive mode. To properly assign the detected *m*/*z* to corresponding (phospho) lipid species, we first analyzed published MALDI-MS analyses of rat BALF for respective phospholipid classes [35, 39, 40]. Next, we compared experimental and theoretical *m*/*z* values from the Metabolomics Workbench Metabolite Database and the LIPID MAPS Structure Database to assign the *m*/*z* detected during MALDI-MS to distinct phospholipid species. To finally confirm the assignments and deduce the composition of the individual fatty acyl residues, MS/MS experiments were conducted regarding distinct *m*/*z*. Phospholipid species were identified via characteristic fragment ions. With this strategy phosphatidylglycerols PG (32:0) and PG (34:1) as well as the phophatidylinositols PI (34:1), PI (36:2), and PI (38:4) were confirmed in the rat lung. It could also be shown by MS/MS experiments (Additional file 1: Figure S2) that PG (32:0) is composed of PG (16:0|16:0). This finding is in accord with a previous study on the composition of BALF phospholipids [40], which also suggests that PG (34:1) is composed of a 16:0 and an 18:1 fatty acyl residue. With respect to PI species the same study on BALF composition showed that PI (34:1) is PI (16:0|18:1), and PI (38:4) is PI (18:0|20:4). For PI (36:2) it was found that it is build up from two 18:1 chains [40]. This finding was also confirmed by MS/MS experiments (Additional file 1: Figure S3), which furthermore identified PI as to be composed of (18:0|18:2). In general, PI species with highly unsaturated acyl residues are highly abundant in BALF and, therefore, seem to be characteristic components of the rodent lung surfactant [40].

Although MALDI-MS is a highly reliable technique one should keep in mind that the detection of distinct phospholipid species is not only concentration-dependent, but also a matter of accessibility to ionization. Intensity differences observed for specific ions likely show differences in amounts of lipids within the sample, however, the final estimation is subject to some limitations [41]_._ More specifically, experimental and theoretical *m*/*z* ratios showed a systematic bias (Δ = − 0.3 Da) possibly caused by the topographical structure of the cryo-section and/or the small height difference of the calibration standard, which had to be pipetted onto the sample target as a small droplet. Notably different starting locations of desorbed ions at the time of acceleration lead to a deviation in the drift time, thus negatively affecting mass resolution. We are aware that all these restrictions make MALDI-MS a semi-quantitative method. Nevertheless, the changes of PI and PG in SiO_2_–n and SiO_2_-FITC NP laden areas, which were imaged with MALDI-MS and confirmed by MALDI MS/MS for the first time, are highly plausible and especially the locally observed increases in PI/PG ratio are in accordance with previous particle-elicited changes of PI and PG in BALF of animals treated with crystalline silica [20].

### Specific changes of phospholipids in the lung

As outlined above, many amorphous SiO_2_ particles and especially crystalline quartz elicit strong inflammation which, in case of quartz, progressively develops into lung fibrosis, accompanied or proceeded by an increased PI/PG ratio [20–22, 42]. The mechanisms underlying these changes in lipid composition are not fully understood. With respect to the local accumulation of PI and other phospholipids (see Figs. 3 and 4) a simple binding to the large surface of deposited SiO_2_-n or SiO_2_-FITC can be ruled out, because phosphonate coating had neither a major effect on polarity or surface charge of SiO_2_-n NP, nor had it an influence on the binding of native surfactant, at least under in vitro conditions [14]. It has been suggested that silica acts on alveolar type 2 cells and induces a switchover in the biosynthesis of phospholipids from the same precursor, thus enhancing PI and suppressing PG synthesis [21]. Further mechanisms may involve a release of ATP from damaged cells followed by an ATP-stimulated secretion by type 2 epithelial cells [43] and/or changes of the activity of specific cleaving enzymes such as phospholipase A2 or phospholipase C [24]. Furthermore, phospholipids are differentially taken up by alveolar type 2 cells and/or macrophages with PI being ingested to a lesser extent both in vitro and in vivo [44, 45]. Based on these studies it appears plausible that more than one mechanism contributes to the local changes of (phospho) lipid concentration elicited by SiO_2_-FITC or SiO_2_-n.

An increased PI/PG increases the rigidity and lowers the surface activity of the surfactant [25] which might be beneficial e.g. for the repair of local tissue damage. Concerning an impact on local inflammation, in vitro experiments suggest that elevated concentrations of PI attenuate the non-specific inflammatory reaction via a reduced production of nitric oxide and tumour necrosis factor alpha (TNF-α) from alveolar macrophages [46]. Considering these findings and the fact that SiO_2_-FITC NPs were mainly localized in alveolar macrophages but not within alveolar type-2 cells led us to suggest that the locally increased ratio PI/PG may originate, at least in part, from the population of NP-affected alveolar macrophages. These cells, when sufficiently loaded with particles, have been shown to release mediators or signalling molecules, which might act on alveolar type-2 cells via specific pathways. This interpretation appears in line with the striking reduction of cytotoxic SiO_2_-n effects on NR8383 macrophages in vitro and the abrogation of inflammation in vivo upon phosphonate coating (see Figs. 1 and 2). A role of macrophages or other inflammatory cells such as neutrophilic granulocytes may also be suspected from a work describing dose-dependent changes of PI/PG in BALF from rat lung upon quartz DQ12: In that study PI/PG developed along with the numbers of cells in BALF, the majority of which were macrophages [20, 47]. In the present study we found that SiO_2_-n and SiO_2_-FITC but not SiO_2_-p led to focal assemblies of macrophages in the lung parenchyma, intermingled with regions of beginning hypercellularity and some structural loss of alveolar septa (Additional file 1: Figure S1). It appears likely that these regions are structural correlates of the patchy areas with increased PI/PG ratio in SiO_2_-n and SiO_2_-FITC treated lungs. Future imaging studies with increased resolution are needed to shed more light on the cellular components involved in particle-induced (phospho) lipid changes in lung tissue.

Apart from the changes in phospholipids there was a decrease in TAG in regions where SiO_2_-FITC was accumulated. Although the final identification of these TAG species awaits further experiments (e.g. tandem MS and high-energy collision induced dissociation and/or high-resolution mass analysis), their presence is highly likely due to the congruent distribution of the DAG-like derivative *m*/*z* 605.7 (Fig. 5) which are derived from TAG in tissue by cleavage of a fatty acyl residue [27]. The local diminution of TAG concentration may reflect its consumption for phospholipid production: TAG is utilized for the formation of dipalmitoyl lecithin, which is the principal lipid in the lung surfactant [48] and which dose-dependently increases in BALF e.g. upon application of quartz to the lung [47]. A lowering of TAG might, therefore, mirror e.g. the new formation of lung surfactant. A decrease of TAG was also found for homogenates of quartz-treated silicotic rat lungs, if values were normalized to the treatment-increased lung weights [48]. As MALDI-MS reports on the concentration of a metabolite in tissue, local decreases of TAG in SiO_2_-FITC laden regions are in line with these findings.

Unlike MALDI-MS, with FT-IR imaging methods molecular information is not generated instantaneously, but through the determination of spectral fingerprints, which can be assigned to particular functional groups in the building blocks of biological tissues, such as lipids, proteins, carbohydrates, and nucleic acids. Besides these building blocks, characteristic biochemical markers of disease are detected and identified. Thus, although FT-IR imaging enables the distinction between healthy and initial-to-advanced states of disease [49], the method has not yet been applied for the examination of nanoparticle-affected lung tissues. Results obtained here appear, however, highly plausible as they showed accumulated lipids, which were confirmed by MALDI-MS as to be mainly related to PI and PG. Furthermore FT-IR spectroscopy revealed a local decrease in protein. Other infrared spectroscopic studies similarly demonstrated an increase in the overall lipid concentration accompanied by a decrease in protein concentration and suggested these changes as suitable markers for cytotoxic [50] or apoptotic changes in cells [51, 52]. With respect to the whole lung, an increase in total phospholipid lung content was observed several days after administration of silica [53], and this effect may involve a transport of de novo formed lipids from liver to lung [48]. As a whole, the locally enhanced lung lipid content in SiO_2_-NP laden areas observed here by means of FT-IR analysis appears to be in line with older results. Application of FT-IR spectroscopy therefore may become important in the field of lung toxicology and histology [54].

## Conclusions

In this study, laterally resolved information on changes in phospholipid and distribution upon SiO_2_-n and SiO_2_-p NPs instilled into the rat lung were demonstrated for the first time by utilizing two powerful bioimaging techniques. MALDI-MS analysis of rat lung tissue sections revealed a local overexpression of PIs and, to a lesser extent, PGs. The pattern of changes was largely congruent with the distribution pattern of SiO_2_-FITC in the lung tissue. FT-IR analyses of a neighboring cryo-sections combined with a subsequent hierarchical cluster analysis revealed regions representing enhanced lipid content, which corresponded invariably with the PI pattern. Notably, these observations were true only for lungs treated with SiO_2_-FITC and SiO_2_-n nanoparticles, but not for lungs which received SiO_2_-p, or for vehicle-treated controls. This disparity was in excellent accordance with in vitro and in vivo toxicity studies which showed that SiO_2_-n or SiO_2_-FITC but not SiO_2_-p induced cytotoxicity and lung inflammation. As the in vitro binding of major surfactant lipids to SiO_2_-n and SiO_2_-p is virtually equal [14], we conclude that the locally increased PI/PG ratio is primarily due to early cytotoxic effects of instilled SiO_2_-n or SiO_2_-FITC.

In sum, bioimaging revealed spatially resolved tissue remodeling of nanoparticle-affected tissue. The results show that changes in phospholipid composition depend on particle surface coating and are related to the particle distribution in the tissue. Therefore, the complementary use of MALDI-MS and FT-IR imaging is assumed to hold a great potential for the examination of health and disease states in biological tissue samples.

## Methods

### Nanoparticle characterization

Colloidal SiO_2_ NPs_,_ the TPMP-treated modification thereof, and fluorescein isothiocyanate-labeled SiO_2_ NPs were provided by project partners of the NanoGEM project and have been extensively characterized before [13, 55]. Additionally, particle suspensions were investigated with a NanoSight instrument (LM-10, Malvern Instruments Ltd., Worcestershire, UK), equipped with a LM14 laser device (535 nm) and NTA software 2.1 to 2.3. All data are summarized in Table 1. The particles showed a similar size under conditions of in vitro (serum-free F-12 K medium and KRPG buffer) and in vivo testing.

**Table 1:** Particle characterization and properties under study conditions (taken from [55])

	SiO_2_-n	SiO_2_-FITC	SiO_2_-p
Primary particle size (TEM)	5–50 nm	23–30 nm	5–50 nm
Average size (AUC)	19 nm	25 nm	19 nm
Zeta potential, pH 7.4	−39 mV	− 39 mV	− 42.9 mV
BET surface	200 m^2^·g^− 1^	178 m^2^·g^− 1^	200 m^2^·g^− 1^
Crystallinity (XRD)	Amorphous	Amorphous	Amorphous
Morphology	Mostly spherical	Mostly spherical	Mostly spherical
Surface chemistry in XPS	O: 66 at.% Si: 29 at.% C: 4 at.% Na: 1 at.%	O: 63 at.% Si: 29 at.% C: 8 at.%	O: 66 at.% Si: 29 at.% C: 5 at.% Na: 0.5 at.% PO_2_, PO_3_: 0.5 at.%
pH of stock suspension	10.2	8.7	10.8
d_50_ in H_2_O (NTA)	47.5 ± 0.7 nm	n.m.	64.5 ± 10.6 nm
d_50_ in KRPG buffer (NTA)	65.3 ± 7.5 nm	n.m.	60.7 ± 7.8 nm
d_50_ in F-12 K medium (NTA)	73.3 ± 14 nm	n.m.	59.3 ± 8.1 nm
d_50_ in instillation fluid (NTA)	61.5 ± 7.8 nm	51.0 ± 4.2 nm	50.0 ± 17.0 nm

*TEM* Transmission electron microscopy, *AUC* analytical ultracentrifugation, *XRD* X-ray diffraction, *XPS* X-ray photoelectron spectroscopy, *NTA* NanoSight tracking analysis, *KRPG* Krebs-Ringer phosphate glucose, *d*_*50*_ median diameter, *n.m*. not measured

### In vitro toxicity study

The rat alveolar macrophage cell line NR8383 was cultured in 175 cm^2^ culture flasks in F-12 K medium (Biochrom GmbH, Berlin, Germany) supplemented with 15% heat inactivated standardized fetal calf serum at 37 °C and 5% CO_2_. Cell culture testing of SiO_2_ NPs was carried out as described by Wiemann et al. [56]. In brief, NR8383 alveolar macrophages were incubated with ascending concentrations of particles in F-12 K medium under serum-free conditions. Assays were run in triplicates in 96-well plates (with 3 × 10^5^ cells per well) and 3 independent experiments were conducted. Untreated cells were used as negative controls. Macrophage supernatants were analysed for typical signs of inflammation indicated by the release of lactate dehydrogenase, glucuronidase, and TNF-α 16 h after addition of the particles. LDH and Glu activities were expressed as % of the positive control value, which was obtained by adding 0.1% Triton X-100. The concentration of TNF-α was measured using 50 μL of the supernatant from each well for inducing apoptosis in L-929 fibroblasts in the presence of actinomycin D and expressed as % killing activity. To measure the release of H_2_O_2_, cells and particles were prepared in Krebs-Ringer phosphate glucose (KRPG) buffer. Quantitative measurements were carried out in the presence of horseradish peroxidase using resorufin as a detection reagent, which was added for 90 min during application of the particles. In all assays cell-free controls were run in parallel to test for particle interferences with the assays.

### Animal study

Female rats (Wistar strain WU, 200–250 g, Charles River Laboratories, Sulzfeld, Germany) were maintained with a 12 h lights-on lights-off cycle with food and water being provided ad libitum. Animals were housed at least 14 d before the experiments were commenced. All animal experiments were ethically approved by local authorities (LANUV, Dortmund, Germany) and were carried out in the animal facility at the University Clinics of Essen, Germany.

Instillation fluid was prepared using a sterile mixture of 0.9% NaCl (9 parts by volume) and sodium buffered phosphate buffer, pH 7.3 (one part by volume). Phosphate concentration was 1 mmol·L^− 1^ and maintained a pH of the instillation fluid in the physiologic range. SiO_2_-n, SiO_2_-p and SiO_2_-FITC particles were diluted from respective stock solutions to a final concentration of 0.72 mg·mL^− 1^. Particle-free instillation fluid was administered to control animals thus generating vehicle-treated controls. For intratracheal instillation, rats were briefly anaesthetized with isoflurane. A total amount of 0.36 mg in 500 μL instillation fluid was intratracheally instilled per animal using a Penn Century Microsprayer inserted into the trachea under visual control. This mass of particles was considered equivalent to the lung burden obtained for SiO_2_-n upon short term inhalation conditions [13]. After 3 d rats were deeply anaesthetized with a mixture of ketamine and xylazine and sacrificed by bleeding from the *Aorta descendens*. A cannula was inserted into the trachea and, while the left bronchus was transiently closed with a Diefenbach clamp, the right lung was lavaged five times with 3 mL, yielding a total of approx. 14 mL BALF per animal for further analyses. Then the right bronchus was clamped and the left lung was inflated with 3 mL Cryomatrix (Thermo Shandon Ltd., Runcorn, UK). The left lung was then resected, snap frozen in liquid nitrogen, and stored at − 80 °C until further processing.

### BALF analysis

Cells from pooled BALF preparations were collected at the bottom of a centrifuge vial (200 × g, 4 °C, 10 min). The supernatant was centrifuged again and the final supernatant was used for protein determination according to the Lowry method [57]. Final numbers of cells were determined with a coulter counter (model Z2, Beckman Coulter GmbH, Krefeld, Germany) and the proportion of dead cells was determined by trypan blue testing. Differential cell counting was carried out with cytospin preparations stained with May-Grünewald or Giemsa dyes. At least 400 cells per animal were evaluated under the light microscope.

### Preparation of lung tissue for fluorescence microscopy

Transverse sections were cut from the hilar region of the left lung with a cryo-microtome (Microtome Cryostsat HM 500, MICROM International GmbH, Walldorf, Germany). Seven μm thick sections were dried onto glass slides and stored under a nitrogen atmosphere at − 20 °C until further processing. To visualize the distribution of fluorescent SiO_2_-FITC NPs, sections were taken from the freezer, fixed with 4% buffered formaldehyde, rinsed thoroughly in phosphate buffered saline (PBS), and covered with a coverslip using Roti-Mount FluorCare (Carl Roth, Karlsruhe, Germany) to stain cell nuclei with the contained 4′,6-diamidin-2-phenylindol (DAPI). Sections were viewed with an inverted fluorescence microscope (Olympus IX51, Olympus Deutschland GmbH, Hamburg, Germany), equipped with a 20× objective and conventional filter sets for DAPI and FITC. In some cases, an antibody labeling of CD68-positive alveolar macrophages was performed as described [58]. Images were taken with a charge-coupled device camera connected to a Nikon Lucia system.

### Sample preparation for bioimaging

For each group the left lung from one animal was chosen for MALDI-MS and FT-IR imaging analyses; selection was based on the protein concentration of BALF as to be typical for the group. In case of SiO_2_-FITC, selection was also based on the distribution of fluorescence which was regarded typical for an intratracheal instillation. Cryo-sections (8 and 10 μm thick) were prepared as described above. The sections were thaw-mounted on indium tin oxide coated glass slides (for MALDI-MS imaging) and calcium fluoride targets (for FT-IR imaging), respectively. Prior to MALDI-MS imaging analysis, frozen tissue sections were allowed to equilibrate to room temperature in a desiccator for ≥2 h. Samples were subsequently washed by submerging the glass slides in 50 mM ammonium acetate buffer (pH 6.7, 4 °C) 4 times, 5 s each, without agitation, to remove the cryo-compound. After each washing step, tissues were dried in a gentle stream of N_2_. After drying the tissues for ≥15 min under vacuum, matrix deposition was performed using a home-built sublimation apparatus. In a vacuum-sealed and pressure-controlled deposition chamber, 25.5 mg of 2, 5-dihydroxybenzoic acid (2, 5-DHB, Sigmal-Aldrich, St. Louis, MO, USA) were quantitatively vaporized and sublimed onto the tissue at 155 °C and 4.7 Pa forming a homogenous layer (0.23 mg·cm^− 2^) of crystals. Before FT-IR imaging the cryo-sections were thawed in a darkened desiccator for 1 h and subsequently washed three times, 5 s each, in *aqua dest.*, followed by a second drying step in a desiccator. As FT-IR imaging provides direct molecular specific information in a non-destructive way, no application of a special matrix is needed.

### MALDI-MS

MALDI-MS measurements were performed using a MALDI-TOF/TOF mass spectrometer (ultrafleXtreme, Bruker Daltonics, Bremen, Germany) operated in reflectron mode. MSI data were acquired using flexControl software v3.4 (Bruker Daltonics). The attenuator offset of the laser (smartbeam-II, wavelength 355 nm) was adjusted to 65% and the laser fluence was set to 45%. For lipid analysis, the mass range was set from 440 to 1700 Da with ion suppression for analytes below 340 Da. Imaging data were acquired in the negative ion mode with a lateral resolution of 50 μm by summing up 100 shots per array position (without intra-spot rastering) using a laser repetition rate of 1 kHz. The sample originating from the rat instilled with SiO_2_-FITC NPs was subsequently rastered in the positive ion mode. Extraction voltage was set to 17.95 kV and lens voltage to 7.50 kV. Mass spectra were calibrated externally using the cubic enhanced algorithm on singly charged ions of bovine cardiolipin disodium salt (Sigma-Aldrich, St. Louis, MO, USA). Data acquisition and image representation were carried out with flexImaging software v3.0 (Bruker Daltonics). Acquired imaging data were normalized to the total ion current (TIC). Lipid identification was based on the comparison of the experimental with the theoretical *m*/*z* values according to the Metabolomics Workbench Metabolite Database and the LIPID MAPS Structure Database (www.metabolomicsworkbench.org and http://www.lipidmaps.org, both provided by the University of California, USA). MS/MS experiments in LIFT mode were performed for selected mass values to confirm structural assignments.

### FT-IR imaging and hierarchical cluster analysis

An infrared hyperspectral image of the lung tissue sample after intratracheal instillation of SiO_2_-FITC NPs was acquired using a Bruker Hyperion 3000 FT-IR microscope system equipped with a liquid nitrogen cooled single point mercury cadmium telluride (MCT) detector. Spectra were collected in transmission mode using 15× Cassegrain objectives. The sample was analyzed by automated raster scanning as a tile mosaic image with a spatial resolution of 100 μm, defined by the aperture and the step size. At every measurement position (area of 100 × 100 μm^2^), an infrared spectrum consisting of 4 accumulations (scans) was collected. The total measured area was 7.1 × 6.6 mm^2^. All spectra were recorded in the range of 400 to 4000 cm^− 1^ with 4 cm^− 1^ spectral resolution. A background spectrum was measured on the CaF_2_ slide outside the tissue sample. Collected spectra were divided over background and automatically converted into absorbance by OPUS 7.0 software. Further spectral pre-processing (baseline correction, scaling, and standardization) and multivariate data analysis were performed with ImageLab software (v.1.94, Epina GmbH, Pressbaum, Austria). Baseline correction applied to spectra was based on the Lieber algorithm in 30 iterations through a 3rd order polynom. Spectral data was scaled between 0 and 1 and standardized (mean = 0.0, standard deviation = 1.0). Prior to hierarchical cluster analysis, spectra with poor signal-to-noise ratio (areas outside the sample) or those which were expected to exhibit properties significantly differing from the lung tissue under investigation (bronchus, bronchioles and blood vessels) were eliminated. These so-called “bad pixels” were excluded from further statistical evaluation. HCA was performed using Ward’s method, Euclidean distance measure and 13 spectral descriptors consisting of important spectral features (proteins, lipids) for identifying tissue remodeling due to nanoparticle instillation.

### Statistical evaluation

In vitro data were generated in triplicates and at least three independent repetitions were carried out. To test for significant differences in vitro, values from each concentration were compared to the non-treated controls using 2-way ANOVA with Dunnett’s multiple comparisons test. In vivo experiments were carried out with 5 animals per group. BALF cell data were compared pair-wise to the corresponding control group for both AM and PMN by 2-way ANOVA with Dunnett’s multiple comparisons test, BALF protein data were compared pair-wise to the corresponding control group by one-way ANOVA and post-hoc Dunnett’s multiple comparison test. A value of *p* ≤ 0.05 was considered significant (*). All data are expressed as mean ± standard deviation (SD).

## Additional file

Additional file 1: **Figure S1.** Effect of different SiO_2_ NP on lung histology. **Figure S2.** MALDI-MS/MS spectrum resulting from the fragmentation of precursor *m*/*z* 721.4. **Figure S3.** MALDI-MS/MS spectrum resulting from the fragmentation of precursor *m*/*z* 861.5. **Figure S4.** Ion images from a vehicle-treated control lung. **Figure S5.** Ion images from a SiO_2_-p-treated control lung. (DOCX 1889 kb)

## Abbbreviations

AM: Alveolar macrophage
BALF: Broncho-alveolar lavage fluid
DAG: Diacylglyceride
DAPI: 4′,6-diamidin-2-phenylindol
EDTMP: Ethylenediamine tetra (methylene phosphonic acid)
FT-IR: Fourier transform infrared (microspectroscopy)
HCA: Hierarchical cluster analysis
m/z: Mass-to-charge ratio
MALDI-MS: Matrix-assisted laser desorption/ionization mass spectrometry
NP: Nanoparticle
PG: Phosphatidylglycerol
PI: Phosphatidylinositol
PLC: Phospholipid composition
PMN: Polymorphonuclear leukocytes
PS: Phoshatidylserine
SiO_2_: Silica
SiO_2_-FITC: Fluorescent SiO_2_ (core labelled with fluorescein isothiocyanate)
SiO_2_-n: Pristine form of SiO_2_
SiO_2_-p: TPMP coated (phosphonated) SiO_2_
TAG: Triacylgyceride
TPMP: 3-(tri-hydroxysilyl) propyl methyl phosphonat
