# Supplementary material for: Phosphonate coating of SiO2 nanoparticles abrogates inflammatory effects and local changes of the lipid composition in the rat lung: a complementary bioimaging study
Source: Part Fibre Toxicol. 2018 Jul 16;15:31. doi: 10.1186/s12989-018-0267-z (PMC6048815; doi:10.1186/s12989-018-0267-z)
Supplement: Supplementary file 1 — Figure S1. Effect of different SiO2 NP on lung histology. Figure S2. MALDI-MS/MS spectrum resulting from the fragmentation of precursor m/z 721.4. Figure S3. MALDI-MS/MS spectrum resulting from the fragmentation of precursor m/z 861.5. Figure S4. Ion images from a vehicle-treated control lung. Figure S5. Ion images from a SiO2-p-treated control lung. (DOCX 1889 kb) [file 12989_2018_267_MOESM1_ESM.docx]

**Supporting information**

**Phosphonate Coating of SiO_2_ Nanoparticles Abrogates Inflammatory Effects and Local Changes of the Lipid Composition in the Rat Lung: A Complementary Bioimaging Study**

Mandy Großgarten,^1*^ Matthias Holzlechner,^2*^ Antje Vennemann,^3^ Anna Balbekova,^2^ Karin Wieland,^2^ Michael Sperling,^1^ Bernhard Lendl,^2^ Martina Marchetti-Deschmann,^2^

Uwe Karst,^1^ and Martin Wiemann^3^

^1^ University of Münster, Institute of Inorganic and Analytical Chemistry, Corrensstraße 28/30, 48149 Münster, Germany

^2^ TU Wien, Institute of Chemical Technologies and Analytics, Getreidemarkt 9, 1060 Vienna, Austria

^3^ IBE R&D Institute for Lung Health gGmbH, Mendelstraße 11, 48149 Münster, Germany

* These authors contributed equally

Corresponding Author: [martin.wiemann@ibe-ms.de](mailto:martin.wiemann@ibe-ms.de)

Phone: ++49 251 9802340

Fax: ++49 251 9802349


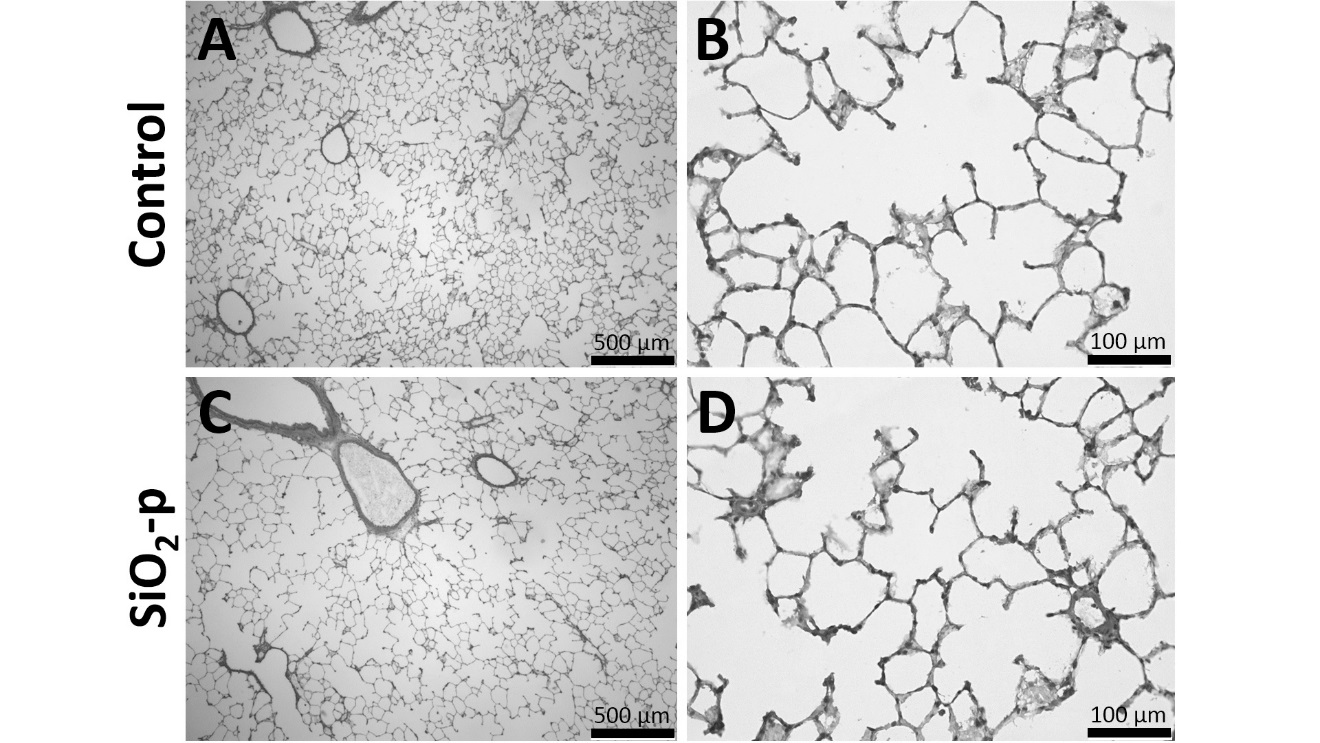


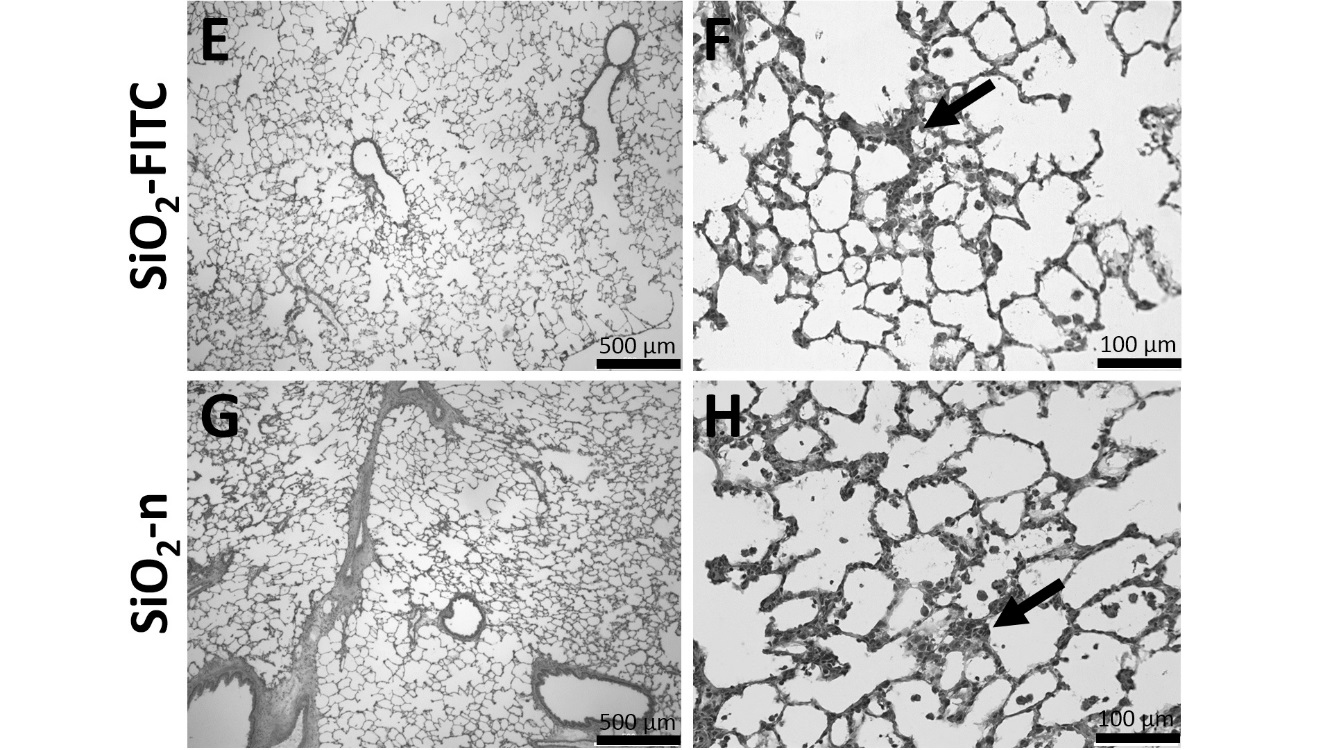


**Figure S1.** Effect of different SiO_2_ NP on lung histology. Typical aspects from lung parenchyma 3 days after intratracheal instillation of 0.5 mL vehicle control fluid (A, B), 0.36 mg SiO_2_-p (C, D), 0.36 mg SiO_2_-FITC (E, F), or 0.36 mg SiO_2_-n (G, H). Hematoxylin-eosin stained cryo-sections. Note that SiO_2_-FITC (F) and SiO_2_ (H) leads to confined regions with increase macrophage numbers, slightly deteriorated septal structures and focal hypercellularity (arrows in F and H). These changes were largely absent in lungs treated with vehicle control (B) or SiO_2_-p (D).

**
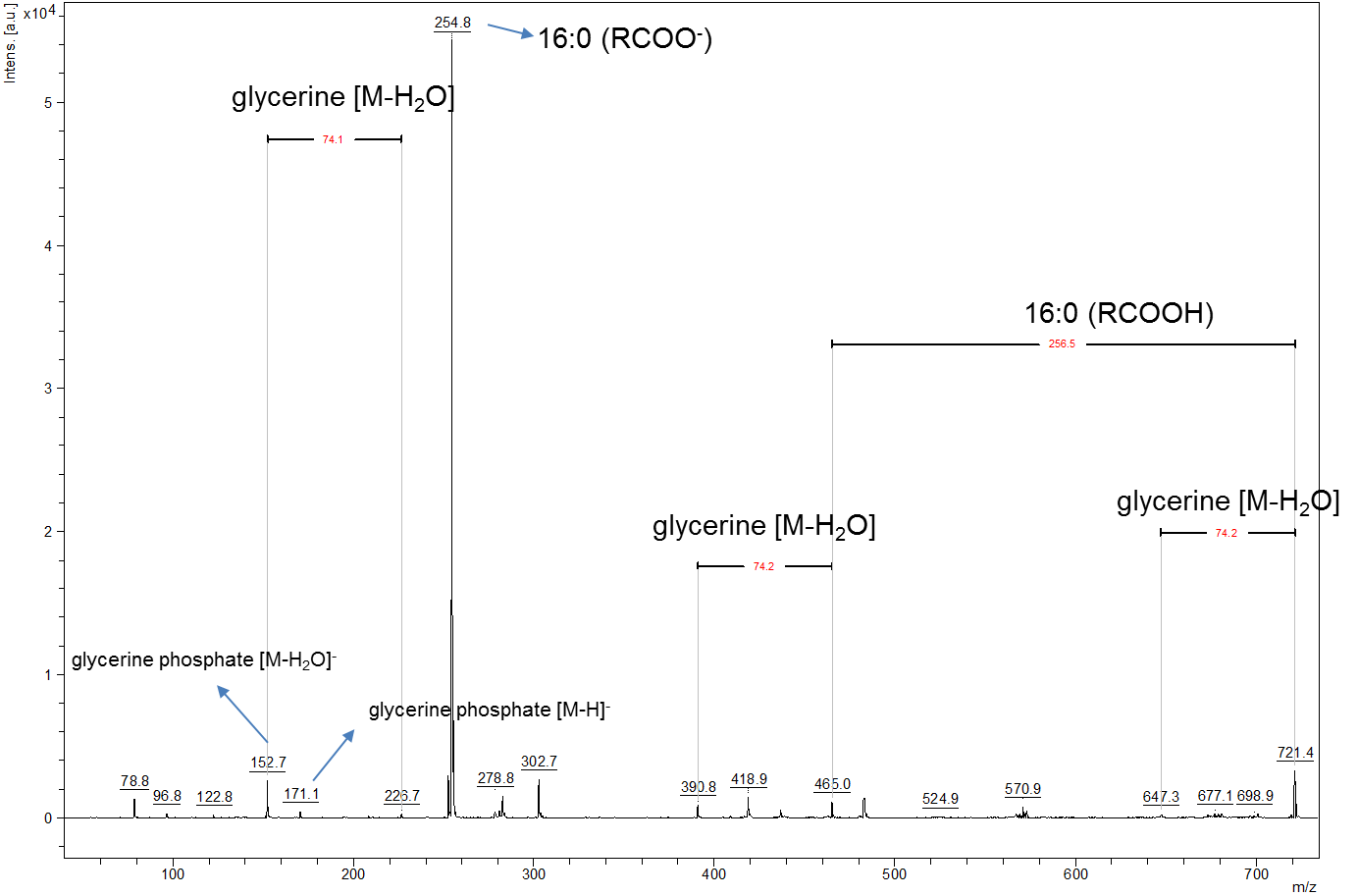
**

**Figure S2.** MALDI-MS/MS spectrum resulting from the fragmentation of precursor *m*/*z* 721.4. Fragment ions confirm the assignment of the respective MS ion image to PG (32:0) and indicate a fatty acyl composition of two (16:0) chains.

**
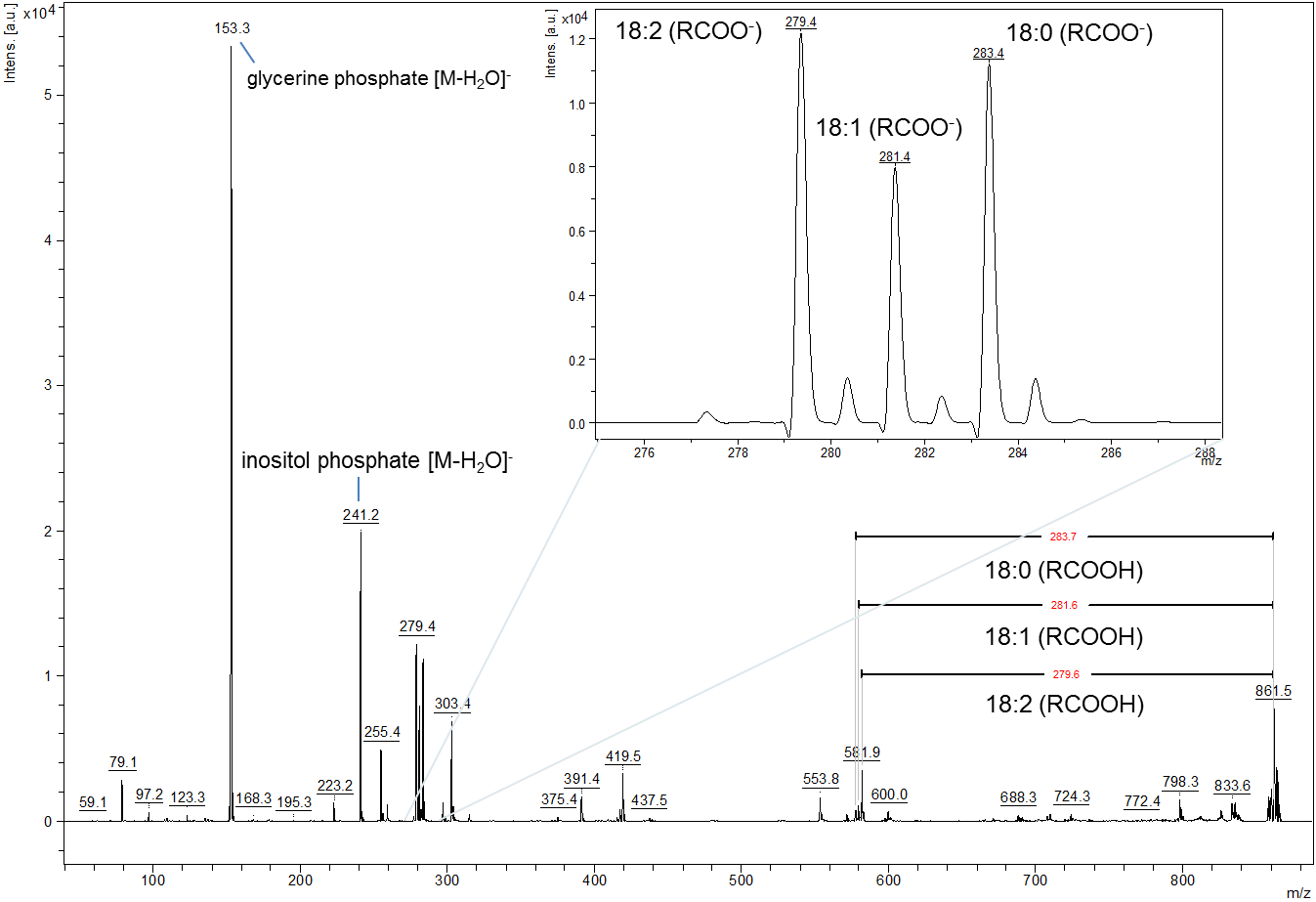
**

**Figure S3.** MALDI-MS/MS spectrum resulting from the fragmentation of precursor *m*/*z* 861.5. Fragment ions confirm the assignment of the respective MS ion image to PI (36:2), whereby both fatty acyl compositions, PI (18:1|18:1) and PI (18:0|18:2) are deduced.

**
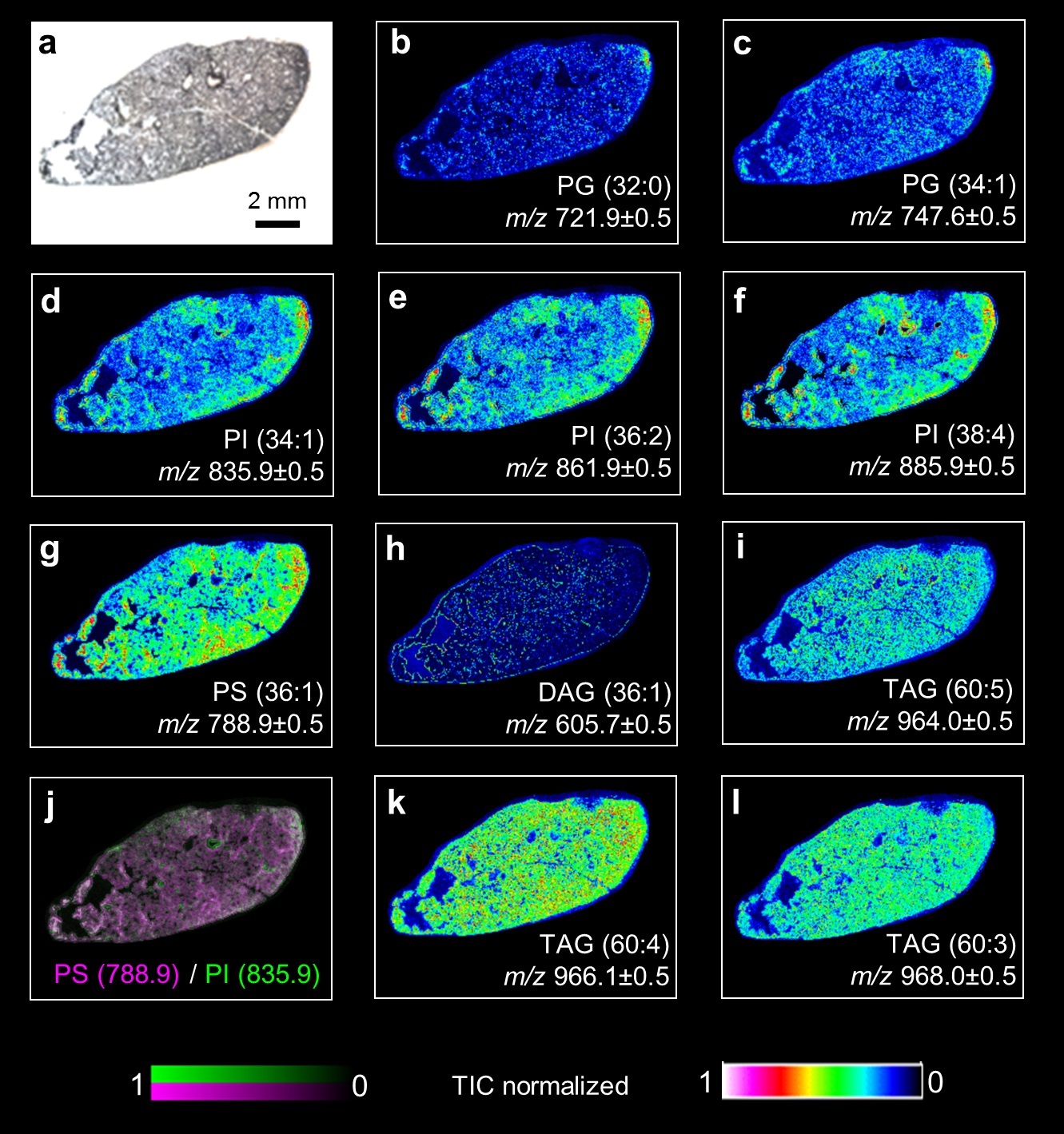
**

**Figure S4**. Ion images from a vehicle-treated control lung. *m/z* values are shown underneath each panel and were measured in the negative ion mode, except *m/z* 605.7 which was measured in the positive ion mode. Data were normalized by division through the total ion current (TIC). (a) microscopic image, (b, c) phosphatidylglycerol (PG), (d-f) phosphatidylinositol (PI), (g) phosphatidylserine (PS), (h) diacylglycerol (DAG)-like fragment, (j) overlay from (d) and (g), (i, k ,l) triacylglycerol (TAG)-like fragments. Note that all analytes are evenly distributed unless compression or damage of the tissue slice, as obvious from (a), has led to an irregular color coding.

**
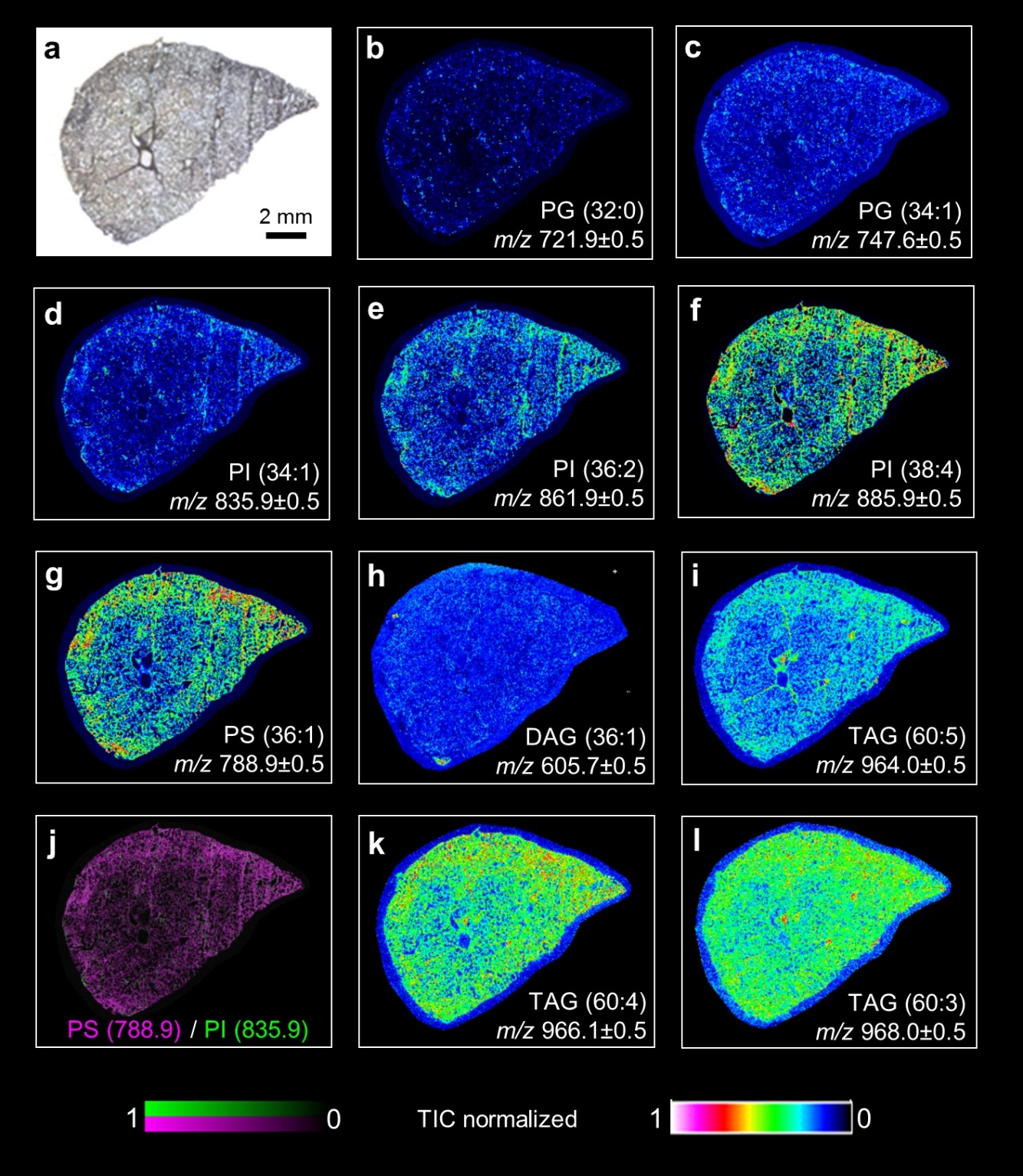
**

**Figure S5.** Ion images from a SiO_2_-p-treated control lung. *m/z* values are shown underneath each panel and were measured in the negative ion mode, except *m/z* 605.7 which was measured in the positive ion mode. Data were normalized by division through the total ion current (TIC). (a) microscopic image, (b, c) phosphatidylglycerol (PG), (d-f) phosphatidylinositol (PI), (g) phosphatidylserine (PS), (h) diacylglycerol (DAG)-like fragment, (j) overlay from (d) and (g), (i, k ,l) triacylglycerol (TAG)-like fragments. Note that all analytes are evenly distributed unless compression or damage of the tissue slice, as obvious from (a), has led to an irregular color coding.
